# Does practice make perfect? Functional connectivity of the salience network and somatosensory network predicts response to mind–body treatments for fibromyalgia

**DOI:** 10.3389/fpain.2024.1245235

**Published:** 2024-09-05

**Authors:** Sonia Medina, Owen O’Daly, Matthew A. Howard, Albert Feliu-Soler, Juan V. Luciano

**Affiliations:** ^1^ Department of Neuroimaging, King’s College London, London, United Kingdom; ^2^ Department of Health and Biomedical Sciences, University of Exeter, Exeter, United Kingdom; ^3^ Department of Clinical & Health Psychology, Autonomous University of Barcelona, Barcelona, Spain; ^4^ CIBER of Epidemiology and Public Health (CIBERESP), Madrid, Spain; ^5^ Teaching, Research & Innovation Unit, Parc Sanitari Sant Joan de Déu, Sant Boi de Llobregat, Spain

**Keywords:** mindfulness, fibromyalgia, rsBOLD, functional connectivity, brain biomarker

## Abstract

**Background:**

Mind–body treatments can improve coping mechanisms to deal with pain, improve the quality of life of patients with fibromyalgia syndrome (FMS), and reduce perceived pain in some cases. However, responses to these treatments are highly variable, the mechanisms underpinning them remain unclear, and reliable predictors of treatment response are lacking. We employed resting-state blood oxygen level-dependent (rsBOLD) functional magnetic resonance imaging (fMRI) to examine changes in brain functional connectivity (FC) following mind–body treatment that may relate to and predict pain relief.

**Methods:**

We recruited patients with FMS who underwent either mindfulness-based stress reduction (MBSR; *n* = 18) or a psychoeducational program (FibroQoL; *n* = 22) and a treatment-as-usual FMS group (TAU; *n* = 18). We collected rsBOLD data, alongside subjective pain, anxiety, depression, and catastrophizing measures prior to and following treatments. We examined behavioral changes and FC changes in the salience network (SN) and sensorimotor network (SMN) and performed regression analyses to identify predictors for treatment response.

**Results:**

The MBSR and FibroQoL groups experienced significant reductions in pain catastrophizing. After treatment, the FC of the sensorimotor cortex with the rest of the SMN became significantly reduced in the MBSR group compared to the TAU group. The FC between the SN and the SMN at baseline was negatively correlated with pain reductions following MBSR but positively correlated with pain reductions in the FibroQoL group. These results yielded large to very large effect sizes. Following MBSR, only for those patients with lower baseline SMN-SN FC, minutes of mindfulness practice were positively associated with clinical improvement (small to medium effect size).

**Conclusions:**

Different mind–body treatments are underpinned by discrete brain networks. Measures of the functional interplay between SN and SMN have the potential as predictors of mind–body treatment response in patients with FMS.

## Introduction

Fibromyalgia syndrome (FMS) is characterized by widespread musculoskeletal pain, cognitive impairment, and psychological comorbidities. Currently, there is no cure for FMS, and debates regarding whether FMS is an inflammatory disease or a central sensitization syndrome remain unresolved (1). Multicomponent treatments are currently the best strategy for managing chronic pain symptoms (2). These include psychological and mind–body interventions (3) alongside usual care to alleviate the emotional burden arising from FMS. Only in some cases do these practices reduce pain (4). FMS is likely the result of a complex interaction between poor stress regulation, perturbed pain control mechanisms, and genetic predispositions (5), resulting in altered nociception without tissue damage. This taxonomy is known as nociplastic pain (6).

Mind–body interventions, however, yield highly variable results (7), making it challenging to predict treatment response, likely due to incomplete knowledge of their mechanisms of action. Mindfulness-based techniques rely primarily on learning to shift the attention from incoming stimuli from one's body to the environment, and vice-versa. Only in some cases is there a resultant reduction in perceived pain. A recent review (8) suggested that the anterior cingulate cortex (ACC) and anterior insula (AI) play key roles in mindfulness-related enhancement of cognitive control and body awareness, yet the precise mechanism to achieve so remains unclear. The ACC and AI are also the main nodes of the salience network (9), which assigns relevance to sensory input to be processed and acted upon (10, 11). During evoked pain testing, meditators also experience greater activity in somatosensory regions, i.e., prime areas of the sensorimotor network (SMN), as subjective pain is reduced (12, 13). However, the involvement of the SMN at rest in meditators with FMS has yet to be explored.

Abnormalities in regions within the SN and SMN at rest are among the most commonly reported findings in patients with FMS compared to healthy controls (14). However, it remains unclear how the SMN and SN interact with each other in relation to treatment response in FMS. Resting-state blood oxygen level-dependent (rsBOLD) functional magnetic resonance imaging (fMRI) is a well-suited technique for exploring relationships between brain networks at rest, referred to as functional connectivity (FC) (15). FC perturbations have been described in persistent pain conditions, and FC relationships can predict responses to drugs for FMS (16, 17). Nevertheless, the predictive potential of rsBOLD in response to non-pharmacological interventions for FMS is yet to be described.

In this study, we aimed to identify FC changes following two different mind–body treatments, with the intention of understanding similarities and differences in their mechanism of action. The treatments examined were mindfulness-based stress reduction (MBSR) and FibroQoL, a psychoeducational program [for a general description of FibroQoL, see Pérez-Aranda et al. (18)]. We examined FC changes following interventions as adjuvants of treatment as usual (TAU), compared to TAU only. We hypothesized that (1) the FC between SN and SMN would decrease following treatment, as effective appraisal of sensory input by the SN is reestablished; (2) clinical improvement (measured through pain, pain catastrophizing, anxiety, and depression scales) would be greater in the MBSR and FibroQoL groups compared to the TAU-only group; and (3) the FC of the SN and SMN would predict symptom changes following MBSR and FibroQoL treatments.

## Methods

### Experimental subjects

From a total sample of 180 who participated in the main randomized controlled trial (RCT), 90 women (30 patients per treatment arm) with FMS were recruited from the Rheumatology Service at Parc Sanitari Sant Joan de Déu, St. Boi de Llobregat, Spain, for the neuroimaging component of this study. The inclusion criteria included the following: being female, having an FMS diagnosis according to the 1990 American College of Rheumatology Criteria, being between 18 and 65 years of age, being right-handed, being able to understand Spanish, and being able to provide written consent.

Patients were excluded if they were participating in other RCTs; had cognitive impairments, comorbid mental disorders, or severe medical illnesses; were receiving psychological treatment; were experienced meditators; were unable to attend group sessions; or presented contraindications for MRI scanning. Additional exclusion criteria prior to each MRI scanning visit included consuming more than eight daily caffeine units (one caffeine drink was permitted on the day of the study) or smoking more than five cigarettes per day. Acute pain unrelated to FMS, such as headaches, was also an exclusion criterion. The participants were not allowed to take any rescue analgesics other than their TAU for 72 h prior to each MRI session to avoid any potential confounding effects.

### Study design and procedure

The present study was part of a 12-month, parallel-group, randomized, single-blind, controlled trial with three treatment arms, namely, TAU + MBSR, TAU + FibroQoL, and TAU only. The Consolidated Standards of Reporting Trials 2010 (CONSORT) (19) were followed (Trial Registration: NCT02561416).

**Figure 1:**
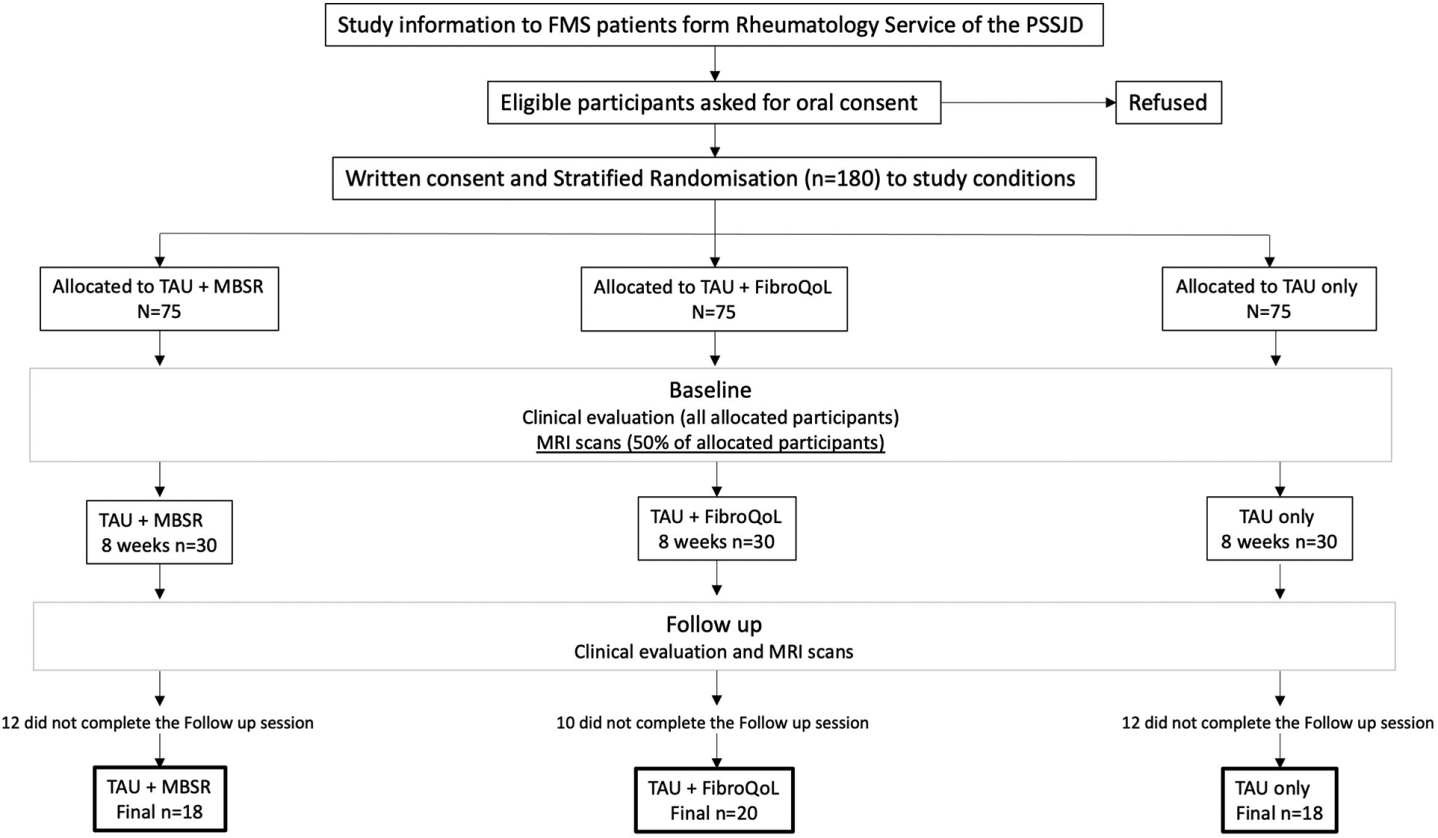
Flowchart of experimental design for the EUDAIMON neuroimaging rsBOLD substudy. Figure adapted from EUDAIMON study protocol (26). rsBOLD, resting-state blood oxygen level-dependent.

Initially, patients were randomly allocated to one of the three RCT treatment groups (i.e., TAU, TAU + MBSR, and TAU + FibroQoL). Once the allocation was completed, 30 patients of each treatment arm were assigned to the RCT + neuroimaging substudy. The substudy visits took place as follows: (i) baseline visit, where patients, still blind to treatment allocation, undertook clinical assessment with self-report measures and MRI assessments, (ii) 8 weeks of 2 h sessions according to treatment arm allocation, and (iii) posttreatment assessments identical as those at the baseline session. A flowchart of the study design can be found in Figure 1, and a full report of the primary outcome of the RCT can be found in Pérez-Aranda et al. (20).

### Interventions

The treatment as usual (TAU) common to all treatment arms included pharmacological treatment and counseling according to patients’ usual therapy plan, which was also complemented by physical exercise where possible. TAU remained consistent throughout the duration of the study. Both interventions (MBSR and FibroQoL) consisted of eight weekly 2 h group sessions (*n* = 15). In each session, patients from the MBSR group received mindfulness training following the MBSR protocol developed at the University of Massachusetts Medical School, USA, that includes practice on body scan, meditation, and contemplation of FMS symptoms from a non-judgemental, accepting perspective, as well as gentle mindful stretches. Patients from this group were also provided with an MBSR book (21) and the option of attending a half-day silent retreat between Weeks 6 and 7. The FibroQoL treatment entailed a purely educational part (first four sessions) where patients received thorough information regarding the pathophysiology and diagnostic criteria of FMS and the main strategies to manage its symptoms and reduce uncertainty and anxiety toward their condition. The last four sessions focused on training in self-hypnosis toward deep relaxation and improved body control, with the objective of gaining control or “escaping” their pain symptoms, imagining their life in the future without pain (in contrast with mindfulness strategies). All patients received audio material to encourage and facilitate further practice at home.

### Materials and measures

We included the following measures in the present study according to *a priori* questions and hypotheses specific to the imaging data. A full summary of all the outcome measures collected in the main clinical trial can be found in the study protocol (22).

#### Demographic, clinical, and self-report measures

The participants provided their age, number of years since FMS diagnosis, and medication as part of their TAU. They also rated their perceived pain intensity on the day of each scanning session using a paper-and-pencil horizontal, 100 mm visual analog scale (VAS), anchored with “no pain” and “maximum pain imaginable” (23). In addition, the patients in the MBSR group were instructed to keep track of their daily minutes of practice at home to have a better idea of how much training each patient carried out. Finally, participants’ scores in the fibromyalgia impact questionnaire—revised (FIQR) (24), the hospital anxiety and depression scale (HADS) (25), and the pain catastrophizing scale (PCS) (26) obtained in each session were included in the analysis set to assess the overall severity of FMS symptoms across the sample.

#### MRI data acquisition and preprocessing

Imaging was performed on a 3.0 T Philips Ingenia wide-bore MR scanner, equipped with an eigh-channel, phased-array, receive-only head coil. On each session, all patients had a T1-weighted 3D structural scan via a turbo field echo (TFE) pulse sequence with 233 slices, repetition time = 11 ms, echo time = 4.9 ms, flip angle = 8°, field of view = 240 × 240 × 174.75 mm, and voxel size = 0.75 × 0.75 × 0.75. The patients also had a perfusion fMRI scan, the results of which have been reported elsewhere (27), and one functional rsBOLD fMRI scan per session, consisting of a single-echo, echo planar imaging sequence (repetition time = 2,000 ms, echo time = 25 ms, flip angle = 90°, matrix size = 80 × 79, in-plane resolution = 3 mm, field of view = 240 mm, 40 transverse slices acquired in interleaved order with thickness of 3 mm and no gap, 240 volumes). For image preprocessing, we used the FMRIB Software Library (FSL) (2012) version 5.0.11. First, we employed FSL FEAT to perform 3D volume realignment of the functional images using MCFLIRT, skull stripping of both functional and T1 scans with BET, and spatial smoothing of the time series data with an 8 mm full-width at half-maximum Gaussian filter. Then, we estimated the coregistration parameters of the mean functional volume to the structural images in native space by employing affine linear registration using FLIRT and the normalization parameters of the T1 scans to a template via non-linear warping using FNIRT. We then proceeded to de-noise rsBOLD images by adopting an independent component analysis approach using ICA-AROMA. Following this, the time series from white matter (WM) and cerebrospinal fluid (CSF) signals were extracted and regressed out from each functional image via a general linear model (GLM). We performed additional motion correction by calculating the framewise displacement time series for each image, using scripts available as part of the BRAMILIA toolbox (https://users.aalto.fi/∼eglerean/bramila.html); volume-to-volume displacement of >0.5 mm was regressed out from the time series. To eliminate sources of noise contained at low frequencies, we carried out high-pass filtering of the functional images (200 s). For normalization to Montreal Neurological Institute (MNI) space, we applied affine and non-linear warping parameters estimated earlier to the functional scans using trilinear interpolation.

### Self-report data analysis

To ensure that the three treatment arms were comparable at baseline, we performed a one-way analysis of variance (ANOVA) for age, years of FMS diagnosis, and VAS, FIQR, HADS, and PCS scores. We also explored within-group changes in FMS symptoms following treatment via paired samples *t*-tests for VAS and FIQR scores. We performed these analyses using SPSS v.26 (https://www.ibm.com/uk-en/products/spss-statistics). Results from the PCS subscales were corrected for multiple comparisons using Holm–Bonferroni correction (28).

### Neuroimaging data analysis

We adopted a mass univariate general linear model approach for all groupwise statistical analyses of the rsBOLD data. We set all initial cluster-forming height thresholds to *p* < 0.001 and applied family-wise error (FWE) correction at cluster extent *p* < 0.05 for each of our contrasts of interest at the whole-brain level. We also examined results from each contrast within our FC networks of interest (i.e., SN and SMN) via a small volume correction (SVC) for each contrast. The independent SN and SNM masks used for SVC were obtained from the Willard atlas (29).

#### Regions of interest

To examine the FC of the SN, we selected six seeds in the insula cortex, namely, the right anterior, middle, and posterior insula in each hemisphere (30), and two seeds in the left and right dorsal ACC (dACC) (31). For the SMN, we selected areas from its main hubs, including bilateral seeds in the primary somatosensory cortex (SI), secondary somatosensory cortex (SII), primary motor cortex (MI), and supplementary motor area (SMA) (32, 33).

#### FC maps

For each participant*,* we calculated voxelwise Pearson's *r* correlations between the mean time series across seeds from SN and SMN and the remaining voxels in the brain within the gray matter by applying an explicit gray matter mask. Correlation maps were then Fisher *Z*-transformed for group analyses. FC maps were computed in Matlab version 9.5.0 (R2010a).

#### Second-level analyses

**Figure 2:**
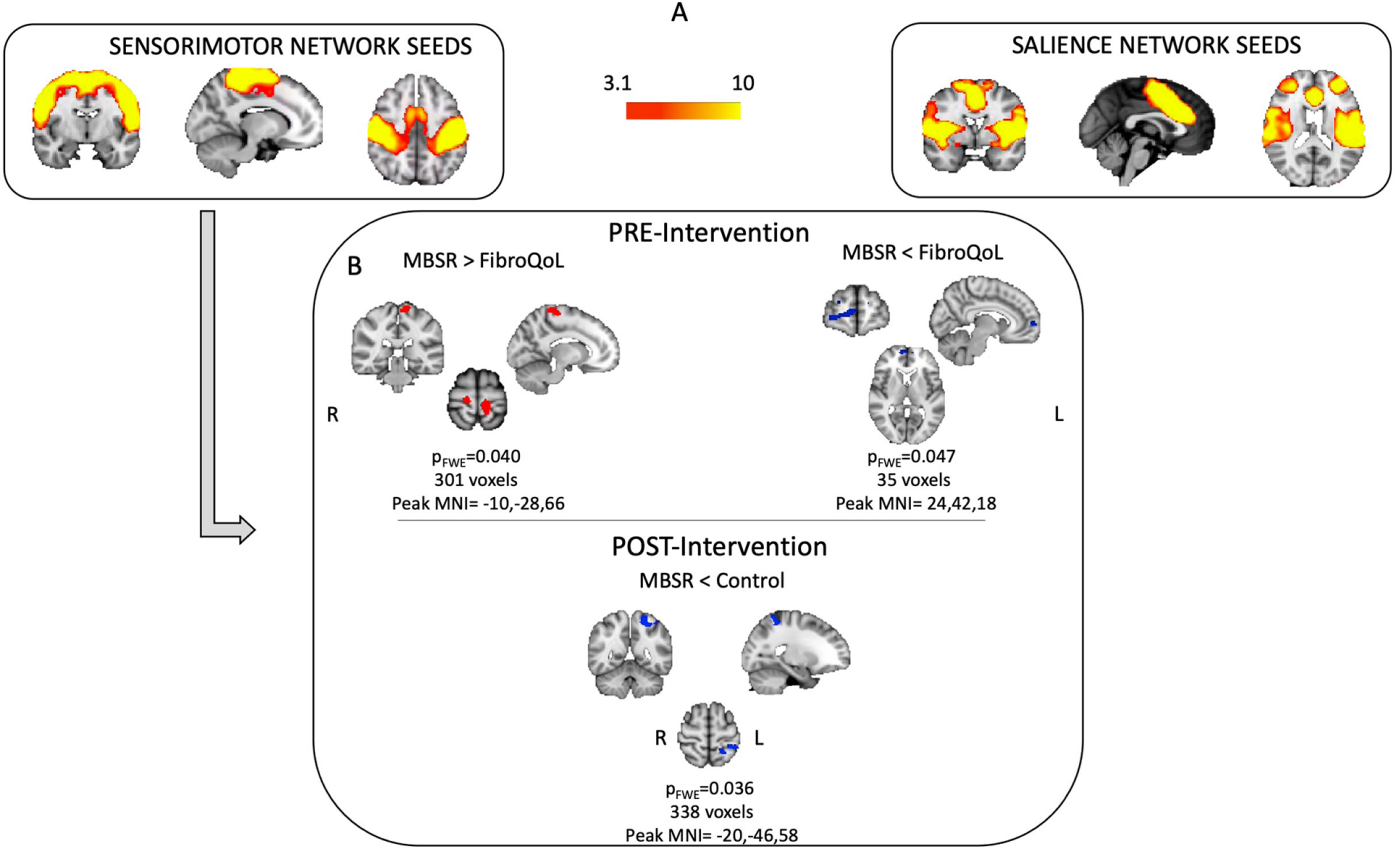
Results from baseline one-sample *t*-test for SMN and SN FC maps **(A)** and results from one-way ANOVA's at baseline and post-intervention using FC from SMN **(B)** FWE, family-wise error; MNI, Montreal Neurological Institute; SMN, sensorimotor network; SN, salience network.

To confirm the correct position of the seeds and accurate distribution of FC within each network, we first performed a one-sample *t*-test across all patients at baseline for both the SN and SMN. Once we verified that the FC of the seeds resembled our FC networks of interest (Figure 2A), we explored treatment-induced FC changes via a mixed 3 × 2 ANOVA with “treatment” as a between-subject factor (i.e., TAU + MBSR, TAU + FibroQoL, and TAU only) and “period” as a within-subject factor (i.e., baseline and posttreatment). We also compared the FC of each network across treatment arms within each session via one-way ANOVAs, with “treatment” as a between-subject factor. We assessed whether baseline FC related to FMS pain symptoms via a multiple regression model across all participants with baseline VAS as a regressor. Finally, we assessed the potential of baseline FC as a marker of treatment outcome via a multiple regression model within each treatment arm and each network separately, taking delta VAS (baseline and posttreatment VAS scores) as a regressor. To maximize the sensitivity of all regression analyses, we calculated the number of spikes (i.e., the total number of volume-to-volume displacements >0.5 mm) and the cumulative distance traveled (i.e., sum over the framewise displacement vector) per image and added them to the second-level models as additional nuisance variables. Given the complex and multifaceted nature of FMS and the resulting potential associations between FC and various factors, alone or simultaneously, including age, duration of the disorder, symptom severity, symptom variety, concurrent medication, and comorbid disorders, we refrained from incorporating these variables as nuisance regressors into our models. This decision was guided by the need to mitigate the risk of overfitting the models, leading to the risk of modeling spurious relationships and poor generalisability, especially when dealing with limited sample sizes (34). Similarly, the use of covariates to deal with potential confounding effects involves high statistical complexity in the context of group difference analyses (35). All models above were performed in SPM 12 (36).

Based on the results obtained from our regression analyses in the MBSR group, we further explored minutes of practice as a predictor of treatment outcome as a function of baseline SN FC. To do this, we first calculated “practice × baseline FC” interaction maps, by multiplying demeaned minutes of practice by demeaned FC at each voxel. We then built a regression model using FSL GLM with two standard regressors (overall mean and demeaned minutes of practice), two voxelwise regressors (i.e., demeaned baseline FC maps of the SN and “practice × baseline FC” interaction maps), and delta VAS as the dependent variable. We focused on the negative correlation between interaction maps and delta VAS, further to our previous results. We employed permutation-based inference using FSL randomize, using a threshold-free cluster enhancement (TFCE) adjusted alpha of *p* < 0.05 to infer significant clusters. Spatial variance smoothing (sigma = 3.4 mm) was employed (37).

#### Effect size

Since *a priori* sample sizes for this neuroimaging substudy were calculated primarily based on evidence on achieved power from perfusion fMRI studies (38), we computed *a posteriori* voxelwise effect size map from all our main contrasts of interest. We calculated an approximation of Hedge's *G* (*G*_a_) statistic from each resulting voxelwise T map (39). Hedge's *G* provides an unbiased estimation of effect size over other measures such as Cohen's *d*, especially in sample sizes below 20 (40).

## Results

### Self-report clinical measures

Out of the 90 patients, 34 patients withdrew prior to completion of the follow-up session (Figure 1). A total of 56 patients were included in the final treatment-by-session analyses (*n* = 18 in TAU + MBSR, *n* = 20 TAU + FibroQoL, and *n* = 18 in TAU). Baseline one-way ANOVAs across our self-report measures of interest revealed that, although initial samples on treatment arms were age-matched, there was a significant effect of age in the final sample (*F*_(2) _= 3.521, *p *= 0.037). Specifically, the TAU-only group was significantly younger than the MBSR group (*p = *0.01). The MBSR and FibroQoL groups did not differ in age. There was no significant effect of the treatment arm for years since FMS diagnosis (*F*_(2) _= 2.204, *p *= 0.122), baseline subjective pain scores (*F*_(2) _< 1, *p *= 0.551), or FIQR scores (*F*_(2) _= 1.855, *p *= 0.167). Baseline vs. follow paired *t*-test within each treatment group revealed significant reductions in total PCS scores, magnification subscale scores, and helplessness subscale scores in the MBSR group and significant reductions in total PCS scores and rumination PCS scores in the FibroQoL group. No significant differences between baseline and follow-up pain VAS scores, FIQR, and HADS measures were observed across groups. These data are presented in Table 1.

**Table 1:** Descriptive statistics (M, SD) in self-report measures and results from paired *t*-test analyses across treatment groups.

	TAU + MBSR		TAU + FibroQoL		TAU	
	Baseline (*N* = 18)	Post (*N* = 18)	Baseline (*N* = 20)	Post (*N* = 20)	Baseline (*N* = 18)	Post (*N* = 18)
Age	56.33 (7.56)	–	54.3 (7.45)	–	49.76 (7.42)	–
Years from FMS diagnosis	13.8 (8.92)	–	10.5 (4.36)	–	8.06 (8.85)	–
Pain VAS (0–10)	5.33 (2.44)	4.92 (2.02)	6.16 (± 2.01)	5.45 (2.28)	5.85 (2.07)	5.15 (2.09)
*t* (sig)	0.587 (0.56)		1.72 (0.100)		0.84 (0.41)	
FIQR (0–100)	56.85 (20.71)	48.87 (21.34)	66.01 (16.99)	60.90 (20.65)	54.18 (23.87)	52.19 (25.08)
*t* (sig)	1.584 (0.13)		1.767 (0.09)		0.593 (0.56)	
HADS depression (0–21)	6.55 (4.61)	6.50 (4.48)	9.15 (5.12)	9.30 (6.02)	7.35 (4.75)	6.94 (4.54)
*t* (sig)	0.04 (0.96)		−0.162 (0.87)		0.35 (0.73)	
HADS anxiety (0–21)	9.72 (3.92)	8.50 (5.37)	11.30 (3.57)	10.70 (4.05)	9.11 (4.27)	9.11 (4.03)
*t* (sig)	1.04 (0.30)		0.711 (0.48)		0 (1.00)	
PCS total (0–52)	18.41 (15.28)	13.29 (10.57)	25.90 (12.58)	22.55 (12.61)	17.56 (10.87)	17.18 (12.15)
*t* (sig)	**2.25** **(0.03)**		2.02 (0.057)		0.143 (0.88)	
PCS rumination (0–16)	5.52 (5.17)	4.29 (3.75)	9.75 (4.77)	7.85 (4.08)	6.68 (4.20)	6.31 (4.15)
*t* (sig)	1.55 (0.13)		**3.11** (0.006)*		0.40 (0.69)	
PCS magnification (0–12)	4.35 (3.58)	3.0 (2.47)	4.70 (2.73)	4.50 (± 2.91)	3.5 (2.52)	3.37 (2.70)
*t* (sig)	**2.14** (**0.04)**		0.33 (0.74)		0.23 (0.81)	
PCS helplessness (0–24)	8.52 (7.36)	6.0 (5.46)	11.45 (6.39)	10.20 (6.49)	7.37 (5.30)	7.50 (± 7.14)
*t* (sig)	**2.26** (**0.03)**		1.5 (0.15)		−0.07 (0.94)	

Not all variables had the same number of missing cases. FibroQoL, psychoeducational program; FIQR, fibromyalgia impact questionnaire revised; HADS, hospital anxiety and depression scale; MBSR, mindfulness-based stress reduction; PCS, pain catastrophizing scale; TAU, treatment as usual; VAS, visual analog scale. *α* = 0.05.

*Only the PCS rumination subscale in the FibroQoL group remained significant after the Bonferroni correction was applied.

Bold values mean statistically significant (*p* < 0.05).

### Effects of treatment on FC

Mixed 3 × 2 ANOVA did not yield significant results for either FC network of interest. Baseline one-way ANOVA for the SMN FC maps differed between groups. The MBSR group displayed greater FC between SI bilaterally and the rest of the SMN, compared to the FibroQoL group (*t *= 4.48, *p*_FWE _= 0.029, peak MNI coordinates = −10, −28, 66); the FC between SN and the right ventromedial PFC (vmPFC) was also lower in the MBSR group compared to the FibroQoL group [*t* = 4.33, (SVC corrected) *p*_FWE _= 0.047, peak MNI coordinates = 24, 42, 18] (Figure 2). These differences did not remain following treatment, where we observed reduced FC between the right SI and the rest of the SMN in the MBSR group compared to the TAU-only group (*t* = 4.28, *p*_FWE _= 0.036, peak MNI coordinates = −20, −46, 58). There were no significant differences across groups for the SN FC maps following treatment.

### Relationship between FC and clinical measures

**Figure 3:**
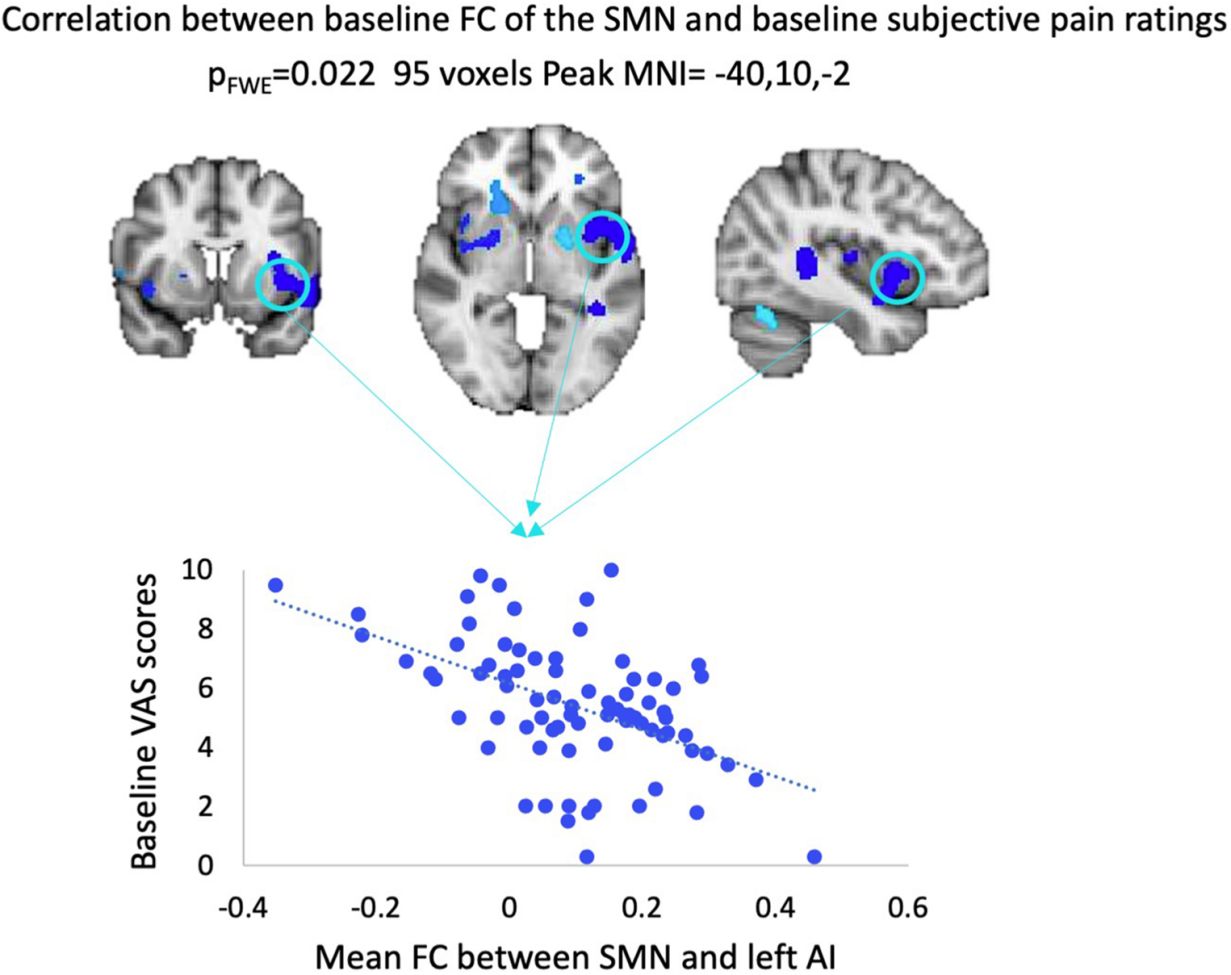
Results from regression analysis between baseline VAS scores and baseline FC of the SMN across all patients. FWE, family-wise error; MNI, Montreal Neurological Institute; VAS, visual analog scale; FC, functional connectivity; SMN, sensorimotor network; AI, anterior insula.

Regression analyses revealed that SN FC did not correlate with VAS scores at baseline, neither across all participants nor within each treatment arm. We also observed that baseline FC of the SMN and left anterior insula (AI) across the whole sample correlated positively with VAS scores, when using SVC within the independently derived SN mask. This correlation did not remain significant while examined within each treatment arm independently (see Figure 3).

### FC as a predictor for treatment response

**Figure 4:**
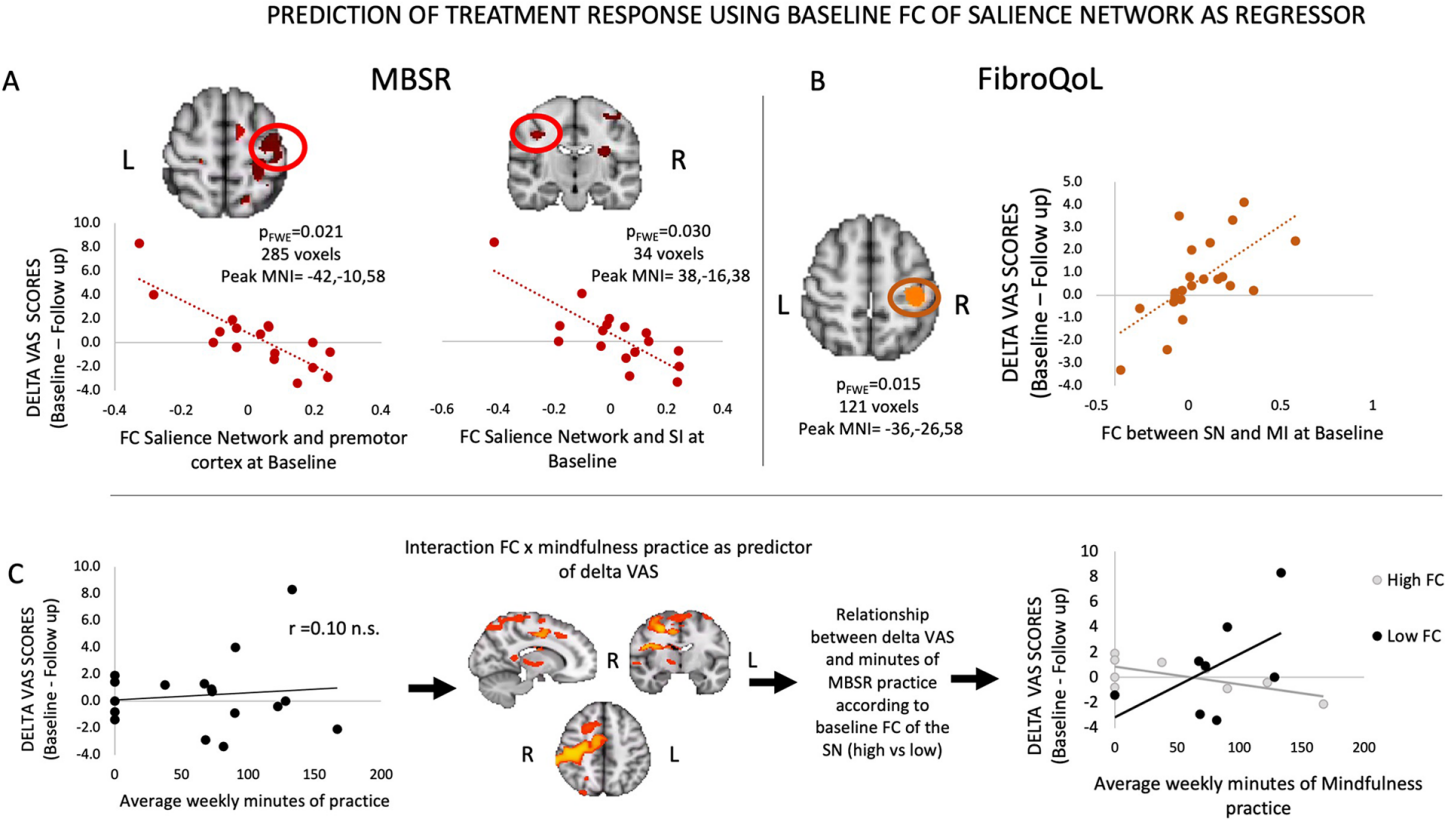
Results from regression analyses between FC of SN and delta VAS in MBSR group **(A)**, FC of SN and delta VAS in FibroQoL group **(B),** and interaction FC of the SN and minutes of mindfulness practice and delta VAS **(C)**. VAS, visual analog scale; FC, functional connectivity; SMN, sensorimotor network; SN, salience network; MI, primary motor cortex; MBSR, mindfulness-based stress reduction; n.s., non-significant.

Our analyses regarding the prediction of treatment outcome indicated that, for the MBSR group, baseline FC of the SN with SI and SII correlated negatively with symptomatic improvement (i.e., delta VAS), which was significant following SVC within the SMN mask (Figure 4). Thus, the greater the FC between these areas at baseline, the smaller the magnitude of improvement in pain symptoms following MBSR. For the FibroQoL group, there was a positive correlation between the SN and the left SI and MI (significant following SVC within the SMN). This finding indicated that greater FC between these areas corresponded to an increase in the magnitude of improvement in pain symptoms at follow-up. Baseline FC within the TAU-only group did not correlate with delta VAS. We did not identify any relationships between treatment-induced outcome and FC of the full SMN when using it as seed.

While examining the correlation between delta VAS in the MBSR group and their “practice × baseline FC of SN” interaction maps, we observed that in a distributed network of areas (i.e., premotor cortex, MI, SI, SII, operculum primary auditory cortex, ventrolateral thalamus), the strength of the interaction of FC and practice was negatively correlated with delta VAS (Table 2). To further interpret these results, we first explored whether there was a linear relationship between minutes of practice and delta VAS. Correlation analyses revealed no significant correlation between both measures (Figure 4C). Following this, we set out to plot the relationship between practice and delta VAS separately for people with *a priori* lower FC between the SN and all significant voxels from the contrast and for people with higher FC. To do this, we calculated the mean FC between the SN and all significant voxels from the contrast. Then we divided the resulting values at the median to obtain two groups: a “low baseline SN-SMN FC” group and a “high baseline SN-SMN FC” group. We observed a positive relationship between practice and delta VAS only in patients with low baseline SN-SMN FC. There was no relationship between practice and delta VAS for patients with high baseline SN-SMN FC. In short, the patients with low baseline connectivity between the two networks experienced larger symptomatic improvement with practice (i.e., the more practice, the bigger the improvement), whereas the patients with high baseline FC did not improve as a function of practice.

**Table 2:** Summary of significant clusters for the negative correlation between interaction maps “baseline SN FC × minutes of practice” and delta VAS in the MBSR group.

Cluster	Side	Peak coordinates (MNI: *x*, *y*, *z*)	Cluster volume (mm^3^)	Peak *t*	*P*_(TFCE)_
SII extending to the SI, MI, premotor cortex, and operculum	Right	46, −14, 24	67,280	5.03	0.011
Middle temporal gyrus	Right	40, −56, 8	6,232	5.01	0.028
Thalamus (MI and premotor projections)	Right	10, −40, 6	2,952	4.73	0.055
Premotor cortex	Left	−38, −16 68	2,304	5.37	0.032

SII, secondary somatosensory cortex; SI, primary somatosensory cortex; MI, primary motor cortex; MNI, Montreal Neurological Institute; TFCE, threshold-free cluster enhancement; MBSR, mindfulness-based stress reduction.

**Figure 5:**
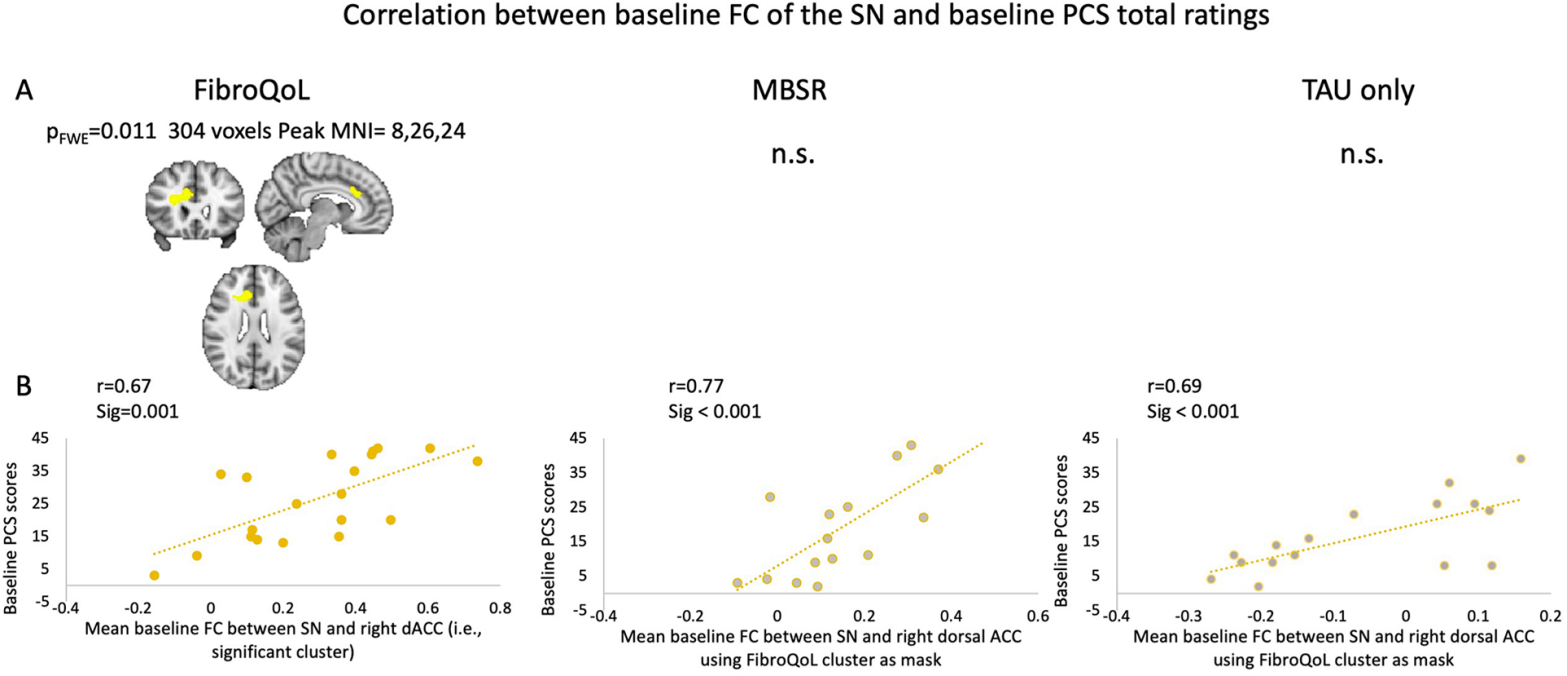
**(A)** Exploratory analysis of voxel correlations between baseline FC of the SN and baseline PCS scores. **(B)** Correlations between FC of the SN and mean FC of the dorsal ACC taking significant cluster from FibroQoL significant cluster as mask. FWE, family-wise error; MNI, Montreal Neurological Institute; n.s., non-significant; MBSR, mindfulness-based stress reduction; SN, salience network; dACC, dorsal anterior cingulate cortex; PCS, pain catastrophizing scale; TAU, treatment as usual.

Due to these results, and since there was a group change in PCS scores in both experimental treatment arms, we investigated whether PCS might contribute to the relationship observed between the FC of the SN and the change in pain symptoms. Within the FibroQoL group, there was a significant positive correlation between baseline total PCS scores and baseline FC of the dorsal ACC (dACC) and the rest of the SN (Figure 5). Similar voxelwise analyses in the MBSR and TAU groups were not significant; however, there was a positive correlation between baseline FC of the SN and mean FC of the dACC when using the significant dACC cluster from the FibroQoL group as a mask from an independent sample, precluding us from incurring in circularity in our analysis (41). There was no significant correlation between baseline FC and delta PCS in all treatment arms, using either total PCS scores or individual PCS subscales as regressors.

## Discussion

We examined functional connectivity changes within SN and SMN following two mind–body treatments to discern insights into their mechanism of action. Contrary to our initial hypothesis, we did not observe within-group changes following treatment; however, the FC changes observed following interventions provide new evidence regarding brain processes underlying each technique. Further, our regression analyses provide important novel mechanistic insights for these interventions. We also provide preliminary evidence of the potential of FC as a predictor of treatment outcome, showing that the FC of the SN at baseline relates to individual differences in treatment response in perceived pain. The direction of this relationship differed by intervention; in the case of the MBSR group, the relationship between weekly mindfulness practice and clinical improvement was positive only for individuals with low baseline connectivity between our networks of interest. In this study, we consider the possible neurophysiological and psychological processes underpinning these findings and propose a model of the general mechanism of action of mind–body treatments for FMS.

Baseline FC between SMN and AI correlated with baseline subjective pain scores when considering the whole FMS sample. Traditionally, the SMN involves stronger FC with the posterior insula (42); however, chronic pain is thought to be characterized partly by impaired integration of stimulus intensity (mediated by sensory regions), salience, and threat attribution, a role primarily played by the AI (43). Accordingly, weaker FC between AI and SMN may lead to poorer perceptual integration, leading to heightened clinical pain experiences. While we did not observe significant within-group reductions in subjectively reported pain after treatment, this is not unexpected. These mind–body techniques aim primarily to modify the appraisal of stimuli, with the ultimate goal of pain control. Therefore, a detachment of negative attitudes towards pain may arguably be expected to arise before group pain reductions are evident. Importantly, it was not our intention to test the efficacy of mind–body treatments to reduce pain in FMS. Rather, we sought to understand their underlying mechanisms and discern the utility of baseline FC as a predictor of treatment responses. The intersubject variability in changes in VAS scores, FIQR, and HADS measures (Supplementary Figure S1) is entirely consistent with clinical reality, as similar symptoms can be underpinned by differential neurophysiological mechanisms that require unique interventions (44). Therefore, it is doubtful that a single intervention would prove effective in treating the majority of the population, and the ability to predict whether these interventions may benefit individual patients before pain becomes refractory to treatment (45) is of utmost importance.

Baseline FC of the SN and the SMN was significantly associated with changes in spontaneous pain following interventions, and intriguingly, the direction of these relationships contrasted between MBSR and FibroQoL groups. The stronger the connectivity between the SN and the premotor cortex at baseline, the less patients benefited from MBSR treatment. The premotor cortex (together with the insula) processes information regarding the perceived unpleasantness of muscle pain (46). It is also involved in the planning and anticipation of actions and also integration of information incoming from the PFC and somatosensory areas (47). Importantly, its activity has also been shown to be associated with pain catastrophizing in FMS (48). We suggest that pain catastrophizing is a key driver of this FC pattern, as well as being the primary target of mindfulness therapies. We also argue that those patients most in need of controlling negative, ruminating thoughts are also the ones most likely to benefit from MBSR.

By definition, MBSR involves continuous training, and so it is reasonable to assume that the more one practices, the more one might improve. Our results, however, suggest that this relationship is hindered in those patients with high baseline FC between the SN and most of the SMN, the middle temporal gyrus (MTG), and the thalamus. The role of MTG in treatment-induced pain improvement is less explicit than those from the areas discussed above. MTG activation may relate to pain anticipation (49), and its integration with areas such as AI and somatosensory cortices has been related to emotional decoding distortions to pain facial expressions (50). Furthermore, its connectivity to PFC and inferior parietal lobule (all areas receiving afferent info from SMN) is associated with auditory verbal hallucinations in schizophrenia (51). Taken together, we speculate that aberrant FC of the MTG with SMN may induce significant distortions in the processing of emotional and somatosensory stimuli that are largely resistant to self-administered mindfulness training. In these patients, improvement is unlikely regardless of the amount of mindfulness practice. The thalamus is a well-known hub in descending pain control systems (38) in addition to its well-known role in afferent signaling (52). A recent review suggested that meditation seems to reduce thalamic activity during pain (53), with the authors suggesting that meditation results in improved ascending pain control in somatosensory and salience networks, facilitating descending pain control via the thalamus. Ineffective communication between ascending and descending pain systems might not preclude some patients from benefitting from mindfulness generally, but it may provide only a limited impact on their pain despite continued practice.

We observed the opposite relationship between FC and clinical improvement in the FibroQoL group, which ostensibly may be considered unexpected. However, FibroQoL involves self-induced relaxation based on hypnosis, a technique thought to modulate motor function via training on motor imagery (going to one's “safe place”) (54). This approach is opposite to mindfulness foundations, which are based on observing and accepting symptoms as they are, rather than seeking “escape” from them. Our findings support the working hypothesis that increased FC between the motor cortex and the SN at baseline provides improvements in treatment-mediated SN leading to increased clinical improvement.

We identified that higher catastrophizing scores were associated with higher connectivity of the dACC with the rest of the SN across all groups at baseline. The dACC is a subdivision of the ACC involved in salience processing (55); excessive engagement of the SN, due to a weak inhibitory action from the dorsolateral PFC provoked by catastrophizing thoughts, has been previously hypothesized. Here, we provide important new evidence supporting this theory (56). Although an overall reduction in PCS scores in both active treatments was observed, individual differences in these scores did not relate to baseline FC in the SN or SMN. We suggest that the relationship between pain catastrophizing and FC may rely more directly on other networks, such as the central executive network. We did not define this network *a priori* as of interest but suggest it warrants further investigation in FMS.

While our results provide insights regarding individual differences in treatment response, we did not observe group reductions in FC between SN and SMN within each treatment arm. We suggest that interindividual variability in response to treatment observed within each group (Supplementary Figure S1), and importantly, the variability observed at baseline, where FC of the SMN differed between treatment groups, is a likely contributor to these null findings. Despite attempts to match the groups on clinical measures, data loss from participant withdrawal and data exclusion contributed to baseline differences in age, which may have at least indirectly led to intrinsic baseline FC differences between the TAU and MBSR groups. Nevertheless, FMS is intrinsically heterogenous, in terms of clinical subtypes, likely underlying pathophysiology and the magnitude of impact on patients’ daily lives (57). Any or all of these differences may contribute to distinct FC patterns in the absence of any treatment intervention. Differences might also be driven by patients’ TAU, given that patients in the FibroQoL group were, on average, receiving fewer drugs throughout the trial (Supplementary Figure S1). Nonetheless, medication remained constant throughout the study. Therefore, we interpret FC shifts after treatment as the result of mind–body interventions specifically.

We also did not observe an overall change in FIQR or HADS scores within treatment arms or a relationship between these measures and FC. While previous evidence, including the results from the main trial from which the present subsample was obtained (20), has shown that meditation and relaxation practice is associated with lower FMS impact, anxiety, and depression (58), our sample sizes were admittedly smaller, which might have in turn limited our ability to detect these effects. Despite this, we detected pain catastrophizing decreases in the MBSR and FibroQoL groups, in line with previous findings (59). This further supports the idea that meditative, mind–body treatments contribute to controlling negative attribution of meaning to stimuli and ruminating thoughts, which form pain catastrophizing phenomena (60).

Notwithstanding the final size of our sample, which was considerably smaller than planned, our regression analyses yielded *post hoc* effect sizes that ranged from small to medium in the case of the baseline relationship between VAS and FC to large and very large in the case of the within treatment delta VAS–FC relationships (Supplementary Figure S3). These were measured via Hedge's *G* statistic to avoid artifactually large effect sizes that can occur in small samples, and therefore our results indicate that these outcomes are of an acceptable magnitude and are not to be discarded as pure chance. Future replications are nevertheless necessary to assess the reproducibility of these results.

In summary, we provide novel insights regarding the application of MBSR for the treatment of FMS. Practicing mindfulness promotes the engagement of attentional networks, modulating the processing of sensory input via the SMN, and providing control over catastrophizing thoughts. The following treatment, “top-down” pain control circuitry integrated with these networks, may provide better control over perturbed activity in the SN via connections with the dACC. These effects may provide better integration of sensory and affective aspects of sensory stimuli by the AI, which ultimately encourages clinical improvement in patients with FMS. We also argue that this therapeutic loop depends upon basal characteristics such as minimally effective stimulus discrimination capacity and relatively functional ascending-descending pain pathways. This mechanism is also different from that occurring in hypnosis-based mind–body treatments, such as FibroQoL. Our findings add further weight to claims of the potential of rsBOLD fMRI as a predictor for treatment outcome, which may be implemented in the future at early stages of treatment planning.

## Data Availability

The original contributions presented in the study are included in the article/Supplementary Material, further inquiries can be directed to the corresponding author.
