# Supplementary figures and images for: Does practice make perfect? Functional connectivity of the salience network and somatosensory network predicts response to mind–body treatments for fibromyalgia
Source: Front Pain Res (Lausanne). 2024 Sep 5;5:1245235. doi: 10.3389/fpain.2024.1245235 (PMC11425596; doi:10.3389/fpain.2024.1245235)

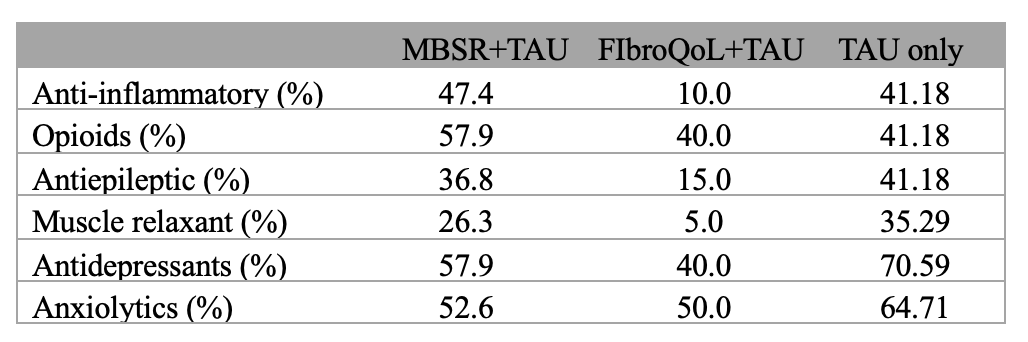

Supplement: Supplementary file 1 [file Image1.jpeg]

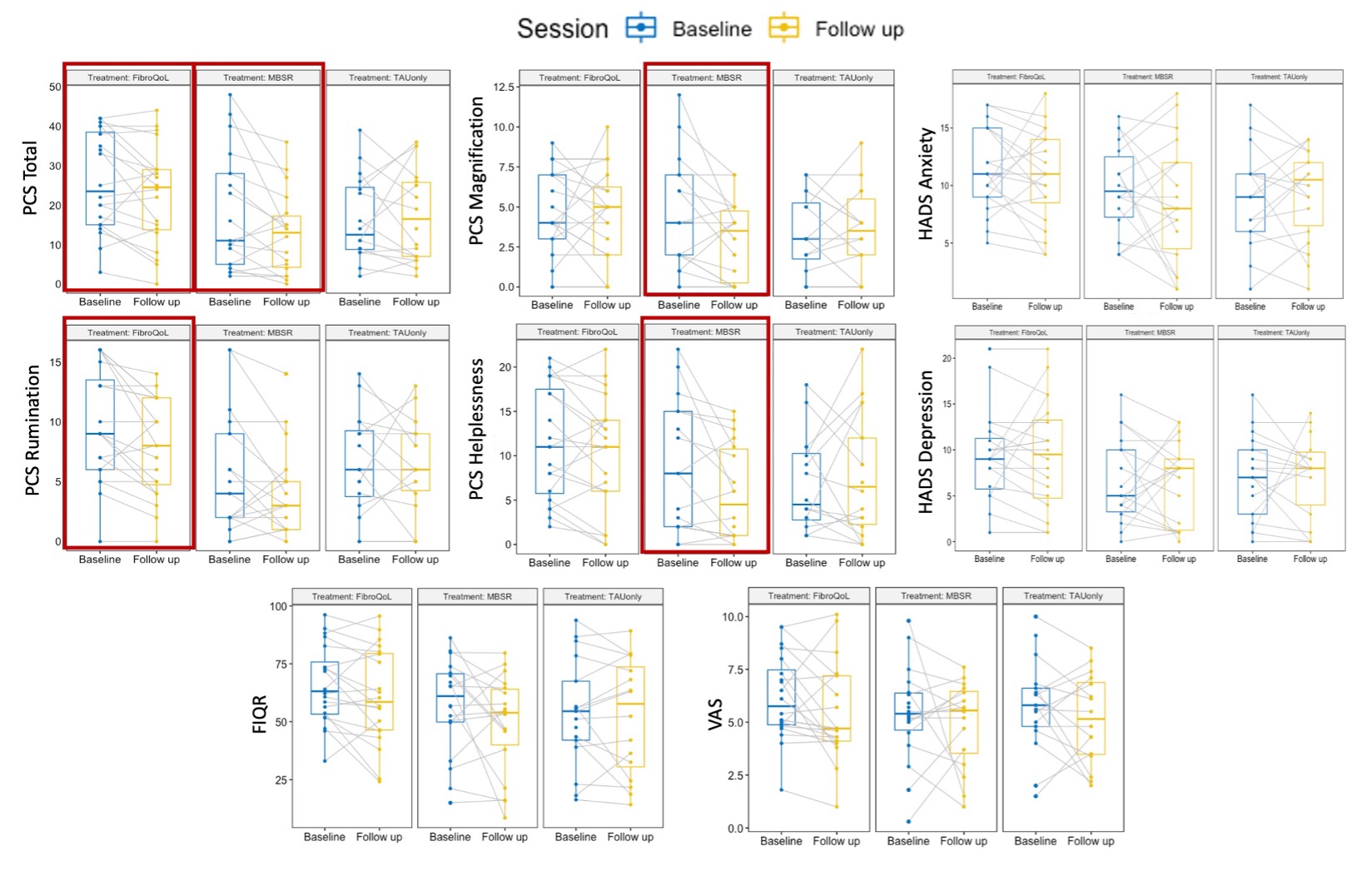

Supplement: Supplementary file 2 [file Image2.jpeg]

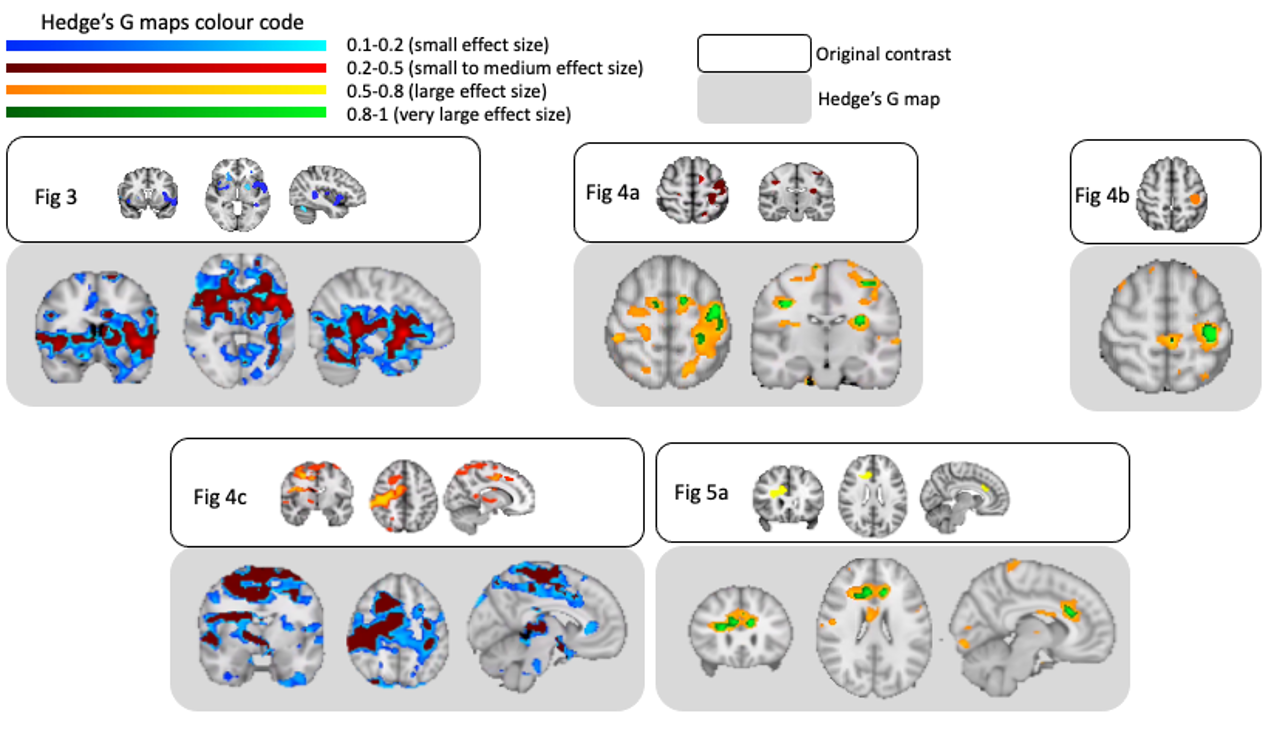

Supplement: Supplementary file 3 [file Image3.jpeg]
